# Brain activity related to individuated finger movement demonstrated by simultaneous motion capture and fMRI

**DOI:** 10.1371/journal.pone.0353360

**Published:** 2026-08-12

**Authors:** Helena Grip, Anna-Maria Johansson, Carl-Johan Boraxbekk, Louise Rönnqvist, Jonas Selling, Charlotte K. Häger

**Affiliations:** 1 Department of Diagnostics and Intervention; Biomedical Engineering, Umeå University, Umeå, Sweden; 2 Department of Psychology, Umeå University, Umeå, Sweden; 3 Division of Health, Medicine and Rehabilitation, Department of Health, Education and Technology, Luleå University of Technology, Luleå, Sweden; 4 Umeå center for Functional Brain Imaging (UFBI), Umeå University, Umeå, Sweden; 5 Department of Neurology, Institute of Sports Medicine Copenhagen (ISMC), Copenhagen University Hospital Bispebjerg, Copenhagen, Denmark; 6 Faculty of Medical and Health Sciences, Institute for Clinical Medicine, University of Copenhagen, Copenhagen, Denmark; 7 Department of Community Medicine and Rehabilitation; Umeå University, Umeå, Sweden; Universita degli Studi di Trento, ITALY

## Abstract

**Introduction:**

The ability to move fingers independently is essential for skilled hand use and relies on both biomechanical constraints and neural control. This study analysed finger independence and associated brain activation during individuated finger movements using simultaneous recordings of 3D kinematics and functional MRI.

**Method:**

Twenty-five right-handed persons (age 62.3 ± 8.2 years) performed flexion-extension movements with individual fingers repeated for each hand during fMRI acquisition. Finger independence was quantified using a 3D motion capture-based Individuation Index (II), and neural activity was analysed with whole-brain BOLD responses. Movement frequency was controlled for in the statistical models. Contrasts between individual fingers and the thumb were used to identify finger-specific brain control regions, followed by region-of-interest analyses of areas commonly activated by fingers with low II. Laterality indices assessed inter-finger differences in hemispheric activation patterns.

**Results:**

II were significantly higher for thumbs (0.98 ± 0.01) and index fingers (0.95 ± 0.03). The middle, ring, and little fingers showed comparable, lower II across both hands, with associations to increased activation in motor-related regions such as contralateral postcentral gyrus, ipsilateral cerebellum and ipsilateral central operculum. Laterality indices decreased with decreasing finger individuation, with significant reductions for the ring and little fingers compared to the index finger in parietal and postcentral regions.

**Conclusion:**

The findings support that lower finger individuation ability is associated with increased recruitment of bilateral sensorimotor and ipsilateral cerebellar regions, even though the underlying mechanisms remain unclear. The integration of kinematic recordings in motor fMRI paradigms may be particularly useful for studying populations with impaired motor control, where variability in movement performance may otherwise confound interpretation of neural activation patterns.

## Introduction

The ability to move fingers independently is essential for skilled hand use and are regulated by both biomechanical factors and neural control mechanisms. The thumb and index finger exhibit the greatest independence [1–3] while the movement of middle and ring fingers cause enslaved movement in adjacent fingers [4]. Finger enslaving is attributed to the mechanical coupling of tendons and muscles that limits the independent movement of these fingers, alongside neuromuscular control mechanisms [3]. The finger movements are controlled by a distributed brain network including the primary motor cortex (M1), where each finger has a partially overlapping but distinguishable representation [5,6]. The network further includes the primary somatosensory cortex (S1), the supplementary motor area (SMA) and the premotor and parietal cortex [7]. The thumb has a more distinct neural M1 representation compared to the other fingers which exhibit more intersecting neural representations [5]. Visualization of the fine grain finger representation in S1 and M1, shows that the fingers have separate and experience-dependent representations in the somatosensory cortex (S1) while multiple, mirrored representations in M1 depend on the movements performed [8–10]. Recent high-resolution fMRI studies, particularly those employing 7T scanners, have further demonstrated fine-grained somatotopic organization of individual fingers within M1 and S1, where the degree of overlap between digit representations often corresponds to lower level of motor precision and independence [10]. A study using multivoxel pattern analysis (MVPA) has demonstrated that distributed activity patterns can reliably differentiate finger movements, despite spatial overlap [11]. This suggests that finger representations are not strictly spatially segregated but are encoded in fine-grained patterns across cortical areas. It is still not fully understood how the brain organizes these overlapping representations across different fingers and the precise contributions and interactions between M1 and the other included regions.

A recent systematic review suggests that combining fMRI with motion capture would offer valuable new insights about cortico-kinematic relationships, though further research is needed to refine processing methods and optimization [12]. One example is however Van Dokkum et al. who studied rhythmic finger movements and found that distinct brain networks were linked to two key performance factors: rhythmicity and error control [13]. In another report, Casellato et al. combined fMRI and kinematic recordings of finger tapping in a stroke patient, showing that incorporating kinematic regressors improved the consistency of brain activation maps [14]. On the other hand, finger individuation has been well described in movement analysis studies, e.g., [15] and [1], where the individuation index (II) is defined to analyse finger individuation: the higher the index, the greater the independence of a finger from movements of the other fingers. Further, simultaneously recorded kinematic data with brain imaging may be used for performance control, which is crucial when analysing fMRI during motor tasks [16].

Movements of single fingers with lower independence place greater demands on motor control compared to more individuated digits such as the thumb, and are therefore thought to involve broader recruitment of sensorimotor regions [17]. In addition, less skilled or less independent movements are expected to require greater neural resources, likely reflecting increased biomechanical constraints, including coupling between adjacent fingers, the need to suppress involuntary movements, and the control of intended movements [5,10].

We therefore hypothesized that fingers with a higher individuation index would exhibit more focal and predominantly contralateral activation in the sensorimotor cortex, whereas fingers with lower individuation would show more widespread and bilateral activation patterns, including increased recruitment of cerebellar regions and ipsilateral sensorimotor cortex.

The primary aim of the current study was to determine the relationship between finger independence and brain activity in right-handed mature adults, using simultaneous recordings of kinematic data and fMRI during a task involving individuated finger flexion-extension movements. The present study focused on right-handed adults with normal hand function and a mean age over 60 years. This population was selected because many clinical populations with impaired hand motor control are in this age range, and establishing baseline measures provides a reference framework for future studies.

## Materials and methods

### Participants

Twenty-six participants with no known previous or current injuries or diseases that could affect the nervous system or the capacity to produce finger movements, were recruited using convenience sampling via advertisements and word of mouth to colleagues and acquaintances. All participants had normal or corrected-to-normal vision and MR-compatible glasses were provided if needed. One participant was excluded due to technical issues with the brain imaging data, leaving 25 participants for further analysis (females n = 13, aged 62.3 ± 8.2 years).

The study was approved by the Regional Ethical Review Board in Umeå, Sweden (Dnr 2011-199-31 M) and was conducted in accordance with the Declaration of Helsinki. All participants gave written and oral informed consent. The data collection was carried out during 2015–2016.

### Task design

All participants performed a task specifically designed to measure each finger´s ability to move independently [15]. All the participants were well acquainted with the task at the time of the fMRI session since they recently performed the same task in a movement laboratory while sitting on a chair, as described previously [2]. All participants were also offered a familiarisation session in an MRI mock-up.

The task was in total performed while the participant was lying supine in the MR scanner with elbows in about 90° of flexion and with both wrists leaning on a custom-made wooden support, keeping their fingers straight and in an extended position. A tilted mirror mounted on the head coil allowed the participant to view task instructions which were presented on a computer screen using both text and illustrative hand images. On the screen, a hand outline (left or right) appeared on a white background, with one finger highlighted in light red. The participant was instructed to perform continuous flexion-extension movements of the instructed finger throughout the 10-second block at a self-selected pace keeping the finger straight and returning it to a fully extended position after each flexion. Movements of the other fingers were to be avoided. The end of each movement block was indicated by a change in the visual display, in which the highlighted finger disappeared. Each trial consisted of five 10-second blocks of finger movement, one block for each finger, presented in a randomized order, and separated by 10-second rest periods to allow the BOLD signal to return to baseline [18]. During the rest period, both hands remained on the supports, with all fingers extended and stationary. The task lasted a total of 8 minutes and comprised six trials, alternating between the right and left hands.

### Data acquisition

The data collection took place at the Umeå centre for Functional Brain Imaging, University Hospital of Umeå, Sweden, which have equipment for combined measurements of kinematics and Magnetic Resonance imaging (MRI). A 3T General Electric MR scanner with a 32-channel head coil was used to acquire structural and functional brain images. A T1 structural image was acquired using the following parameters: 180 slices; 1 mm thickness; repetition time 8.2 msec; echo time 3.2 msec; flip angle 12°; field of view 25 x 25 cm. For the collection of the functional gradient-echo-planar imaging sequence the following scanning parameters was used: repetition time = 2000 msec, echo time = 30 msec, flip angle = 80°, field of view = 25 x 25 cm. Thirty-seven transaxial slices were acquired in an interleaved order, (thickness 3.4 mm, 0.5 mm gap). Ten initial dummy scans were collected and discarded prior to analysis. The graphical instructions were initiated by a sync pulse from the MRI scanner and were programmed in E-prime (version 2; Psychology Software Tools). The computer parallel port was used to also synchronize the programmed instructions with the kinematic data collection.

**Fig 1:**
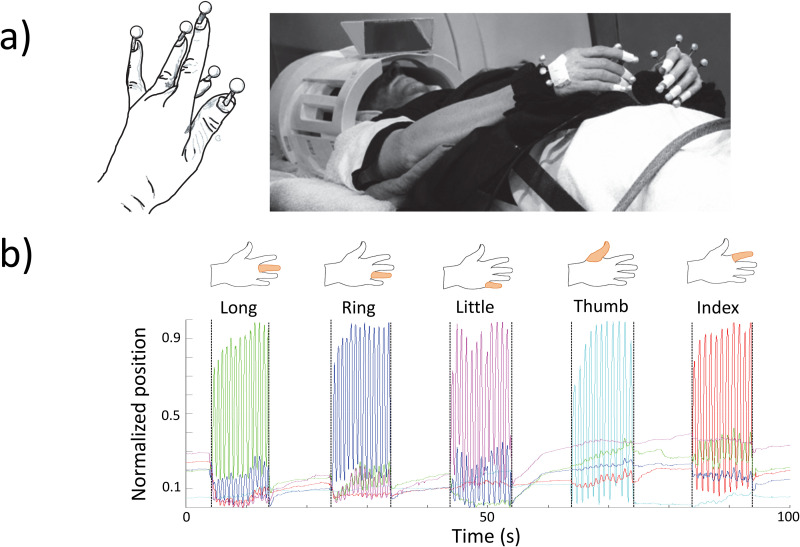
The measurement setup is illustrated in Fig 1a, with the participant lying down and both wrists resting on a custom-made wooden support. Reflective markers attached to the fingertips and the back of the hand, registered by an optical motion capture system. The ten finger markers were used for further analyses. A mirror attached to the head coil was used to provide visual instructions to the participant in the form of a hand, with the instructed finger highlighted in light red as illustrated in Fig 1b together with the recorded 3D finger trajectories. Normalized trajectories were calculated based on the movement slopes of the instructed finger, relative to the enslaved normalized movement slopes of the non-instructed fingers.

Kinematics were recorded with an optical 3D motion capture system (3 Oqus MRI compatible cameras, Qualisys, Sweden) mounted on the walls in the scanner room. Data was collected at 120 Hz and recorded 3D-positions of 7 mm reflective markers. Each marker was attached on a short plastic rod and affixed with double-sided adhesive tape on each fingertip. In addition, a three-marker cluster was placed on the dorsum of each hand but was not included in the analyses (Fig 1).

### Kinematic data processing and analysis

Qualisys Track Manager Software was used to identify 3D marker trajectories, with the gap-fill parameter set to 20 frames. Kinematic parameters were then calculated using Visual3D (Visual3D v5, C-Motion Inc., Germantown, MD, USA) and a custom MATLAB plug-in (MATLAB R2017a, The MathWorks, Inc., Natick, MA, USA). The marker data was first filtered using a 6 Hz 4th-order low-pass zero-phase Butterworth filter.

The block lengths (nominally 10 s) were adjusted based on the kinematic analysis of each finger’s movement onset and offset, identified by tangential velocity profiles for each finger marker trajectory. Each onset was defined as when the tangential velocity reached 5% of the peak tangential velocity, and if the velocity continued to increase until reaching at least 15% of the tangential peak velocity. The offset was defined in a similar way, as when the tangential velocity decreased below 5% of the tangential peak velocity. Events for each peak finger flexion and extension, were automatically identified by local minima and maxima within each block. All events were confirmed by visual inspection of graphs of fingertip trajectory, speed profiles and the animated 3D movements.

Blocks in which the participant moved a finger other than the instructed one or continued performing flexion–extension movements during the rest period, were excluded. Minor unintended movements of additional fingers within a block were expected and considered part of the natural variation captured by the Individuation Index (II) and were therefore retained in the analysis. After these corrections, six movement blocks and one rest block were excluded. A total of 744 corrected blocks remained, with an average block length 9.9 ± 1.4 seconds, and were subsequently used in the first-level fMRI analysis described below.

The *movement frequency*, MF, was calculated as the total number of finger flexions within a block, divided by the total movement time of the adjusted movement block. Time vectors containing MF for each finger and block were calculated and resampled according to repetition time (0.5 Hz, corresponding to 410 samples and 820 seconds).

The normalised positional change of all fingers was used to calculate the *Individuation Index*, II, developed by [1] and adapted from 2D to 3D data as described in detail in our earlier study [2]. II_j_ was calculated for the instructed finger *j* as:


$$\begin{array}{c} {II}_{j}=\text{1}-\frac{\sum_{i=\text{1}}^{n}\left({|S}_{ij}|\right)\;-\text{1}}{\left(n-\text{1}\right)} \end{array}$$

where *n* = 5, and *S*_*ij*_ is the relative motion slope for finger *i* when finger *j* is instructed to move. This gives II_j_ as a number between 0 and 1, where 1 represent perfect independence and 0 represent no independence.

To examine finger-wise differences in the Individuation Index (II) and movement frequency (MF), linear mixed-effects models were fitted with finger (thumb, index, middle, ring, little) as a fixed effect and subject as a random effect (random intercept). Post hoc comparisons between each finger and the thumb were extracted from the model estimates. False discovery rate (FDR) correction was applied to account for multiple comparisons across fingers.

### fMRI preprocessing and analysis

The fMRI data were pre-processed and analysed using the SPM12 software (Wellcome Department of Cognitive Neurology, London, UK) integrated with MATLAB R 2023 b (MathWorks, Inc., Natick, MA). All results are reported using a whole‐brain family‐wise error rate (FWE) correction at the peak voxel level (*p* < .05), with an additional minimum cluster size threshold of 10 voxels to reduce spurious activation. Brain regions were defined and labelled in MNI space using the automated anatomic labelling atlas 3; AAL3 [19,20].

The preprocessing included slice timing correction using the first image as reference slice, followed by movement correction by unwarping and realigning all scans using the first image as reference. A co-registration of the mean functional image series and the structural T1 image set was done, followed by a segmentation of the co-registered T1 image. A Dartel normalized sample-specific template was created based on white and grey matter segments from the segmented, co-registered, T1 images [21]. An affine alignment to Montreal Neurological Institute (MNI) standard space was done. Finally, the images were smoothed with an 8-mm FWHM Gaussian kernel (voxel size was 2 × 2 × 2 mm).

At first level, a subject specific general linear model (GLM) was specified for all individuals. Each experimental condition was modelled as a separate regressor (Finger_i_, i = 1:5, right and left hand, and Rest), using motion-corrected time blocks, with each condition convolved with the canonical hemodynamic response function. The six head motion realignment parameters were included as regressors of no interest. Additionally, an alternative model was evaluated in which each finger’s movement frequency (MF) was included as regressor of no interest, to assess whether movement speed had an effect on the BOLD signal [16,22]. First-level contrasts were computed using t-tests for the ten primary conditions (Finger_i,_ > Rest, i = 1:5, right and left hand).

At the second (group) level, a flexible factorial design with two factors was used, including the factor Subject (25 levels; independent) and the factor Finger > Rest (first-level contrasts Finger > Rest, 10 levels, dependent). Analyses were restricted to a grey matter mask derived from a binarized mean image based on the segmented T1-weighted images of all participants (DARTEL-generated and normalized to MNI space, thresholded at 0.3 and applied as an inclusive mask). No covariates were included in the model. The main effect of Finger_i_ > Rest (i = 1:5, right and left hand), was assessed across conditions. Statistical significance was determined using a cluster-level family-wise error (FWE) correction at p < 0.05.

To assess whether fingers with lower individuation index showed increased ipsilateral involvement in regions of interest for finger movement control a voxel-based, threshold-independent laterality index (AveLI) [23], was computed for the precentral and postcentral gyri, supramarginal gyrus, and superior parietal lobule for each finger condition, using binary masks based on the Neumorphometrics atlas applied on the individual contrast images (Finger_i_ > Rest). Differences between fingers were evaluated using a non-parametric repeated-measures model (Friedman test) followed by post hoc pairwise Wilcoxon signed-rank tests with FDR correction.

### Relationships between BOLD activation and II

Each finger was contrasted against the thumb (Fingerᵢ > Thumb; FWE-corrected, p < 0.05) to identify finger-specific increases or additional recruitment. The thumb served as the reference condition due to its superior individuation ability [1–3].

Fingers exhibiting significantly lower individuation index (II) than the thumb were entered into conjunction analyses (conjunction null; FWE-corrected, p < 0.05), performed separately for each hand, to identify regions commonly engaged across less individuated fingers.

Percent signal change (PSC) was extracted from ipsi- and contralateral regions identified in both the conjunction analyses and the Fingerᵢ > Thumb contrasts. PSC was computed using the MarsBaR toolbox (version 0.44) [24]. For each cluster, the peak voxel was used to define a region of interest (ROI) as a 4 mm radius sphere centered on the cluster maximum. Up to five local maxima per cluster were included to better capture the spatial extent of activation. Voxels outside the original cluster were excluded, as were voxels located in white matter based on the Neuromorphometrics atlas to restrict analyses to grey matter signal. A mixed-effects model was used to test for finger-related effects within clusters identified in the conjunction analysis. Finger was entered as a fixed effect, and participant as a random intercept. Significance of the main effect of finger was assessed using an F-test, with FDR correction applied across clusters (α = 0.05). Where a significant main effect of finger was observed, post hoc pairwise comparisons were conducted comparing each finger against the thumb condition.

Associations between PSC and II across individuals were assessed using Pearson’s correlation coefficient, together with p < 0.05 considered statistically significant. To correct for multiple comparisons, the FDR procedure was applied separately for each finger across all ROIs, and both uncorrected and FDR-corrected p-values are reported.

## Results

Individuated finger movements evoked BOLD responses across widespread cortical and subcortical motor-related networks. Across all movement conditions, activation was consistently observed in the contralateral precentral and postcentral gyri, parietal regions and the ipsilateral cerebellum, with additional involvement of subcortical structures including the putamen and pallidum (Tables S1–S2 in S1 File). Some clusters extended into white matter, likely reflecting partial volume effects or spatial smoothing. Laterality indices (AveLI) showed the expected contralateral dominance for both hands across all regions (Table 1), with positive values for right-hand movements and negative values for left-hand movements. A gradient was observed across fingers, with thumb and index fingers showing consistently higher degrees of lateralisation compared to the middle, ring, and little fingers, indicating reduced lateralisation with decreasing individuation. This pattern reached statistical significance for the left ring and little fingers compared to the index finger in the postcentral gyrus, and for the left little finger compared to the index finger in the superior parietal lobule (Table 2).

**Table 1:** Mean laterality index (AveLI) values [23]for the precentral gyrus, postcentral gyrus, supramarginal gyrus, and superior parietal lobule during right- and left-hand finger movements. Values are presented as mean (SD). Positive values indicate right-hemisphere dominance and negative values indicate left-hemisphere dominance. Significant post hoc comparisons between fingers (non-parametric tests, FDR-corrected) are reported where applicable (*p < 0.05*).

Region of Interest	Precentral gyrus	Postcentral gyrus	Supramarginal gyrus	Superior Parietal lobule
R Thumb	0.35 (0.25)	0.64 (0.20)	0.28 (0.38)	0.36 (0.37)
R Index	0.38 (0.26)	0.69 (0.21)	0.25 (0.46)	0.40 (0.30)
R Middle	0.30 (0.28)	0.63 (0.21)	0.14 (0.39)	0.25 (0.36)
R Ring	0.31 (0.25)	0.63 (0.21)	0.16 (0.37)	0.26 (0.39)
R Little	0.33 (0.23)	0.61 (0.21)	0.16 (0.32)	0.27 (0.36)
L Thumb	−0.37 (0.25)	−0.59 (0.20)	−0.23 (0.34)	−0.44 (0.29)
L Index	−0.43 (0.27)	**−0.63 (0.22)**	−0.19 (0.45)	**−0.52 (0.34)**
L Middle	−0.36 (0.23)	−0.57 (0.22)	−0.22 (0.37)	−0.46 (0.38)
L Ring	−0.32 (0.29)	**−0.50 (0.24)**	−0.22 (0.34)	−0.47 (0.33)
L Little	−0.32 (0.25)	**−0.48 (0.22)**	−0.17 (0.29)	**−0.38 (0.30)**
Post hoc tests		L Ring > L Index (p = 0.047) L Little > L Index (p = 0.035)		L Litte > L Index (p = 0.035)

**Table 2:** Increased brain activation for individual fingers relative to the thumb (Finger > Thumb) on group-level (n = 25 healthy older adults; right-hand dominant) during right (R) and left (L) hand movements. Results are shown at a family-wise error (FWE) corrected threshold of p < 0.05. No significant activations were observed for the right or left index finger compared to the thumb at this threshold and are therefore not reported. Brain regions at local maxima within each identified cluster are listed under “Peak activation” Regions within each cluster are reported and categorized as contralateral and ipsilateral cortical regions, and cerebellar regions (according to Neumorphometrics), excluding voxels located in white matter. “Voxels” give the number of voxels in each cluster, “p” gives the p-value, “T” indicate activation strength and Peak MNI gives the MNI coordinate for each peak activation.

Condition	Peak activation	Contralateral cortical regions	Ipsilateral cortical regions	Cerebellar regions	Voxels	p	T	Peak MNI (X Y Z)		
**R Middle> Thumb**	L Postcentral gyrus	74.9% L postcentral gyrus, 0.6% L precentral gyrus	*N/A*	*N/A*	354	.000	6.30	−44	−32	62
							4.95	−38	−27	53
**R Ring > Thumb**	L Postcentral gyrus	61.7% L postcentral gyrus	*N/A*	*N/A*	222	.000	6.04	−44	−30	62
	L Inferior occipital gyrus	62.5% L occipital fusiform gyrus, 37.5% L inferior occipital gyrus	*N/A*	*N/A*	8	.031	4.83	−24	−96	−12
**R Little > Thumb**	L Postcentral gyrus	75.0% L postcentral gyrus, 3.0% L precentral gyrus, 1.1% L superior parietal lobule, 0.5% L supramarginal gyrus	*N/A*	*N/A*	567	.000	6.90	−44	−30	60
	L Lingual gyrus	88% L lingual gyrus	*N/A*	12% L Cerebellum	25	.018	4.98	−18	−77	−14
	L Superior parietal lobule	100.0% L superior parietal lobule	*N/A*	*N/A*	2	.041	4.70	−29	−57	66
	R Cerebellum Exterior	*N/A*	*N/A*	100% R Cerebellum	3	.039	4.66	20	−53	−23
**L Middle > Thumb**	R Postcentral gyrus	R postcentral gyrus 52.2%	*N/A*	*N/A*	69	.006	6.12	48	−26	57
**L Ring > Thumb**	R Postcentral gyrus	R postcentral gyrus 47.4%	*N/A*	*N/A*	19	.021	5.34	47	−26	57
**L Little > Thumb**	R Postcentral gyrus	R postcentral gyrus 54.8%	*N/A*	*N/A*	126	.002	6.79	47	−27	59

### Finger movement performance

**Fig 2:**
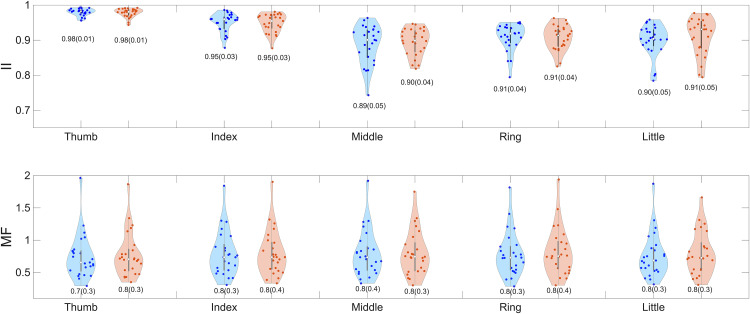
Violin plots showing the Individuation Index (II) and Mean Frequency (MF) for fingers of right dominant hand (blue) and left non-dominant hand (red). Group average and group standard deviation are written below each plot.

II were significantly higher (FDR corrected, p < 0.05) for thumbs (0.98 ± 0.01) and index fingers (0.95 ± 0.03) compared with the other fingers for both right and left hands (Fig 2). The middle, ring, and little fingers showed comparable II across both hands, except for a lower II in the right middle finger compared with the ring finger (p = 0.03).

The mean frequency MF (number of finger flexion-extension movements per second) was on average 0.79 ± 0.03 across all fingers (range 0.29 to 1.96 Hz, Fig 2.). No significant differences in MF were observed between fingers. Models incorporating MF as nuisance regressors in the statistical parametric mapping (SPM) analysis were explored, to investigate if increased speed may give an increase in BOLD signal. At the group level, no such significant differences were observed (model without MF regressors > model with MF regressors), suggesting that movement speed did not substantially influence the second-level results. Consequently, the model without MF regressors was used for further analysis.

### Finger-specific brain activation and its association with individuation index

Movements of the middle, ring, and little fingers were associated with greater activation in the contralateral postcentral gyrus (primary somatosensory cortex) relative to the thumb. Additionally, movement of the right little finger were associated with greater activation in the superior parietal lobule, ipsilateral cerebellum and the occipital cortex (lingual gyrus) when contrasted with the right thumb (Table 2).

**Fig 3:**
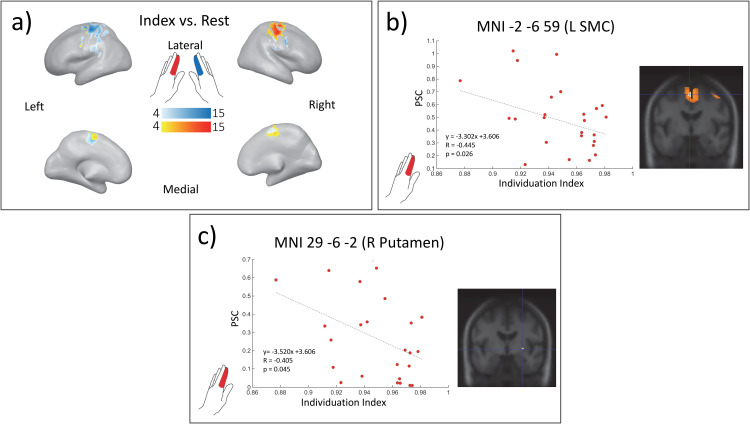
a) Significant group-level activations during individuated Index Finger movements of the right dominant hand (blue) and the left (non-dominant) hand (red), each contrasted against rest (familywise corrected p level < 0.05, cluster size ≥ 10 voxels). Activations are projected onto a canonical FreeSurfer inflated surface (MNI2FS). Horizontal blue and red bars indicate T-values from the SPM analysis. **b–c)** Clusters derived from a conjunction analysis of non-index digits (middle, ring, and little fingers) were used as regions of interest for correlation analyses. Significant correlations were observed between low individuation index (II) and increased activation within a 4 mm radius spherical ROI centered on the peak voxel in the ipsilateral Putamen and sensorimotor cortex (SMC) during Left Index Finger movement. The cluster and spherical ROI (radius = 4 mm) are illustrated on the group-averaged, MNI-normalized T1-weighted anatomical image. Blue crosshairs indicate the peak coordinate.

**Fig 4:**
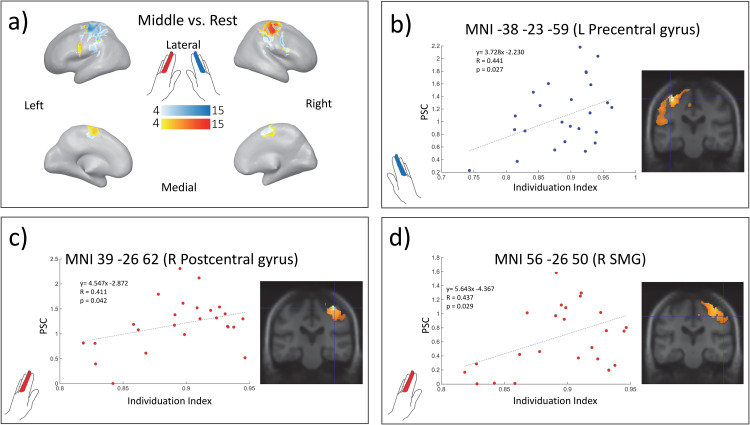
a) Significant group-level activations during individuated Middle Finger movements of the right dominant hand (blue) and the left (non-dominant) hand (red), each contrasted against rest (familywise corrected p level < 0.05, cluster size ≥ 10 voxels). Activations are projected onto a canonical FreeSurfer inflated surface (MNI2FS). Horizontal blue and red bars indicate T-values from the SPM analysis. Clusters derived from a conjunction analysis of non-index digits (middle, ring, and little fingers) were used as regions of interest for correlation analyses. Significant correlations were observed between high individuation index (II) and increased activation within a 4 mm radius spherical ROI centered on the peak voxel in the contralateral precentral gyrus during right middle finger movement (**b**) and contralateral postcentral gyrus and supramarginal gyrus (SMG) during left middle finger movement (**c-d**). The cluster and spherical ROI (radius = 4 mm) are illustrated on the group-averaged, MNI-normalized T1-weighted anatomical image. Blue crosshairs indicate the peak coordinate.

**Fig 5:**
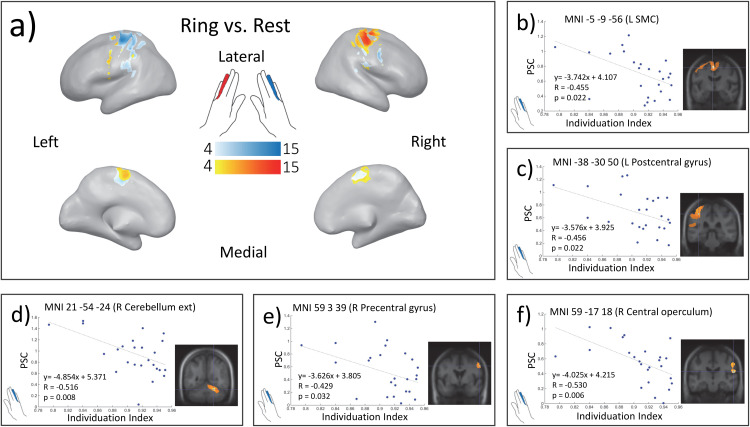
a) Significant group-level activations during individuated Ring Finger movements of the right dominant hand (blue) and the left (non-dominant) hand (red), each contrasted against rest (familywise corrected p level < 0.05, cluster size ≥ 10 voxels). Activations are projected onto a canonical FreeSurfer inflated surface (MNI2FS). Horizontal blue and red bars indicate T-values from the SPM analysis. **b-f)** Clusters derived from a conjunction analysis of middle, ring, and little fingers were used as regions of interest for correlation analyses. Significant correlations were observed between low individuation index (II) and increased activation within a 4 mm radius spherical ROI centered on the peak voxel in the contralateral sensorimotor cortex (SMC) and postcentral gyrus, and ipsilateral cerebellum, precentral gyrus and central operculum during right ring finger movements.

**Fig 6:**
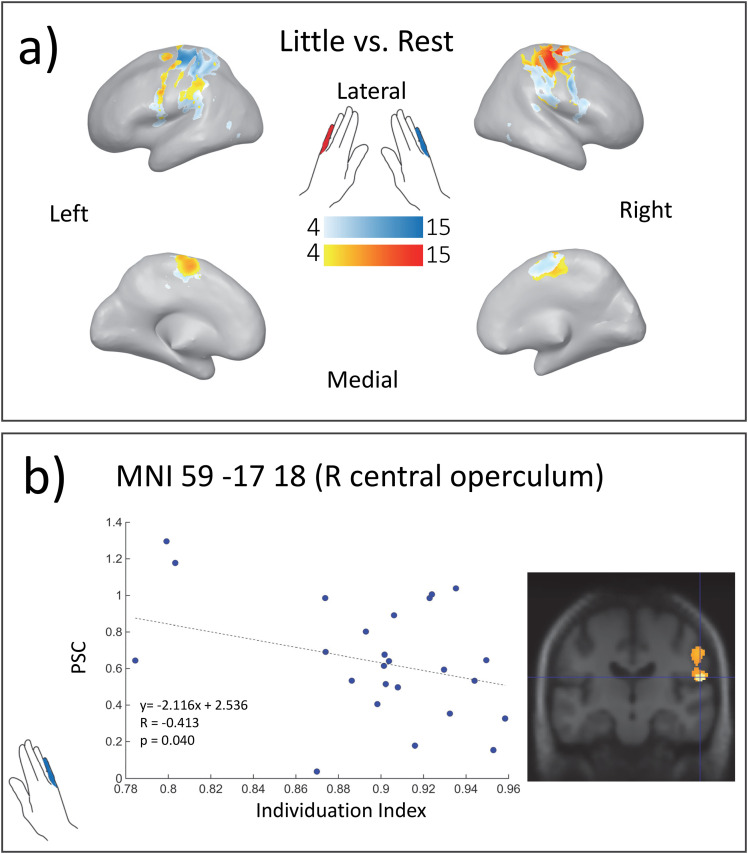
a) Significant group-level activations during individuated Little Finger movements of the right dominant hand (blue) and the left (non-dominant) hand (red), each contrasted against rest (familywise corrected p level < 0.05, cluster size ≥ 10 voxels). Activations are projected onto a canonical FreeSurfer inflated surface (MNI2FS). Horizontal blue and red bars indicate T-values from the SPM analysis. **b)** Clusters derived from a conjunction analysis of middle, ring, and little fingers were used as regions of interest for correlation analyses. Significant correlations were observed between low individuation index (II) and increased activation in ipsilateral central operculum during right little finger movements.

Correlations were assessed between individuation index (II) and BOLD percent signal change (PSC), extracted from spherical (4 mm radius) ROIs commonly activated by fingers with lower II than the thumb (Fig 3-6). Higher finger individuation was associated with increased activation in contralateral sensorimotor regions, including the precentral and postcentral gyri as well as the supramarginal gyrus (Fig 4a–b). In contrast, lower individuation ability was associated with increased activation in the ipsilateral precentral gyrus (Fig 5e), ipsilateral sensorimotor cortex, contralateral putamen (Fig 3c), and ipsilateral cerebellum (Fig 5d). Additionally, lower individuation was accompanied by recruitment of further motor-related regions, including the ipsilateral central operculum (Figs 5f, 6b), contralateral sensorimotor cortex (Fig 5b), and contralateral postcentral gyrus (Fig 5c). After FDR correction, significant associations remained between lower individuation index and increased activation in the ipsilateral cerebellum (Right Ring finger, FDR-corrected p = 0.04) and ipsilateral central operculum (Right Ring finger, FDR-corrected p = 0.03).

A mixed-effects model within clusters defined by the conjunction analysis showed a significant main effect of finger in the left precentral gyrus with the right little finger showing greater responses than the right thumb (MNI −38 −23 59, FDR-corrected p = 0.04). In the left postcentral gyrus right middle, ring and little fingers all showed higher responses than the right thumb (MNI −38 −30 50, all FDR-corrected p = 0.02).

## Discussion

This study aimed to examine the relationship between finger independence and BOLD activation during individuated finger movements. The study focused on older right-handed adults (mean age 62 years), providing a relevant baseline for future studies in clinical populations with impaired hand motor control. The hypothesis was that reduced finger individuation, as quantified by a lower II, would be associated with increased activation in distributed sensorimotor networks.

Consistent with previous behavioural research [1,2], the thumb showed the highest level of independence (0.98 ± 0.01), followed by index fingers (0.95 ± 0.03), while the middle, ring, and little fingers demonstrated lower and comparable independence, see Fig 2. These fingers also exhibited stronger activation in the contralateral primary somatosensory cortex compared with the thumb. This may reflect increased sensory processing demands and a greater reliance on somatosensory feedback in less individuated finger movements, consistent with previous work highlighting the role of sensory input in fine motor control [5,8–10].

In addition, lower individuation ability was associated with increased activation in ipsilateral sensorimotor cortex and cerebellum, suggesting greater involvement of distributed motor control networks during less independent finger movements. While these findings indicate a relationship between reduced finger independence and broader neural recruitment, the underlying mechanisms remain unclear. Possible explanations include increased biomechanical constraints and a greater need to suppress involuntary co-activation of adjacent fingers, or differences in motor control strategies.

### Relationships between finger independence and BOLD activation

At the group level, movements of the middle, ring, and little fingers were associated with greater activation in common regions of the contralateral postcentral gyrus (primary somatosensory cortex) relative to the thumb. We further found that higher II was associated with increased activation in contralateral sensorimotor regions (right middle finger: MNI −38, −23, −59; left middle finger: 39, −26, 62). We additionally analysed laterality indices in sensorimotor regions, supramarginal gyrus and superior parietal lobule, indicating lower laterality (more bilateral activation) in fingers with lower independence. This pattern may reflect more focal activation in fingers with high independence, and on the other hand greater reliance on bilateral sensory feedback during movements of less individuated fingers, consistent with increased demands on maintaining movement specificity. It may also relate to differences in motor representations, as the thumb is thought to have a more differentiated and functionally specialized representation in the primary motor cortex [5].

We found associations between low II and increased activation in sensorimotor cortex (right ring finger, left index finger), ipsilateral central operculum (right ring and little finger), ipsilateral precentral gyrus (right ring finger), contralateral putamen (left index finger) and contralateral SMG (left middle finger). These regions are part of the broader motor network, including premotor, parietal, basal ganglia, and opercular areas, consistently recruited during tasks with higher motor demands [17] and during early stages of motor learning [25]. Increased activation in these regions has been linked to greater requirements for sensorimotor integration, action selection, and movement monitoring, particularly when movements are less automatized or require greater control. In the present context, their recruitment in association with lower finger independence likely reflects increased demands on coordinating and differentiating finger movements, rather than increased neural efficiency.

Additionally, movements of the right little finger had increased activation compared with the thumb in the ipsilateral cerebellar lobule VI region (MNI 20, −53, −23). Within this region of interest (MNI 21, −54, −24) lower finger independence was significantly associated with increased activation for the right ring finger. Conjunction analysis further showed that this region was consistently engaged during movements of the right hand’s middle, ring, and little fingers. Similar activation pattern have been reported in a 7T fMRI finger-tapping study, where random-sequence tapping increased cerebellar engagement, likely reflecting higher demands on motor response selection [26]. Moreover, this region showed stronger activation in musically naive individuals compared to skilled musicians during a bimanual finger-tapping task, suggesting greater motor efficiency in musicians for bimanual coordination [27]. Given the cerebellum’s established role in motor coordination and fine-tuning, increased ipsilateral activation may reflect greater demands on coordination, compensatory recruitment, or increased task difficulty [28].

The dominant finger movements activated a more widespread network including the ipsilateral superior temporal sulcus, contralateral cerebellum and the contralateral supplementary motor area, with more correlations to finger individuation than for left hand’s fingers. It could reflect that dominant and non-dominant sides are specialized for different aspects of motor control. For example, the dominant arm is more specialized in controlling speed, movement direction, and timing, while the non-dominant arm is more specialized in impedance control [29] and outperform the dominant arm in tasks that lack visual feedback about hand position [30].

To summarize, fingers with lower individuation were indeed associated with more widespread and bilateral activation patterns, including increased recruitment of cerebellar regions and ipsilateral sensorimotor cortex. This pattern is consistent with greater demands on coordination and motor control when moving less independent fingers. However, the present data do not allow us to determine whether these effects reflect increased motoric effort, biomechanical constraints, differences in motor strategies, or compensatory neural recruitment. Importantly, correlational analyses may also imply individual differences in motor skill [28], rather than task-driven differences alone, which cannot be disentangled in the current design. Consequently, these findings should be interpreted as reflecting an association between finger independence and neural recruitment, rather than a causal mechanism.

### Influence from finger movement frequency on BOLD response

The fMRI protocol was not specifically designed to examine the influence of finger speed on the BOLD signal, instead participants were instructed to move fingers at a self-selected preferred speed to optimise or at least not compromise the control of independence. Notably, the work of Oliveira et al. (25), demonstrated a linear increase in the BOLD response with movement frequency, with a plateau at movement rates exceeding 1 Hz [22], and the movement frequency in our study (0.93 ± 0.05 Hz) was within the range observed to elicit a significant BOLD response. We did not observe any frequency-dependent effects on the fMRI data on group level (model without MF regressors > model with MF regressors), suggesting that the self-selected finger speed did not markedly influence the results. This may be more relevant to account for when comparing individuals with impaired finger control to those without such impairments, where differences in movement speed could contribute more noticeably to the BOLD response.

### Strengths and limitations

By simultaneous registration of kinematics and fMRI, we were able to identify correlations between low finger independence and increased activation in cerebellum. This multimodal setup also facilitated the correction of block timing inconsistencies, exclusion of erroneous data, and control for movement speed, thereby minimizing potential confounds. This approach may be particularly valuable when analysing individuals with impaired motor control or those who have difficulty following task instructions, such as those with neurological conditions [12].

Finger-specific differences in BOLD activation were not strongly differentiated at the level of peak activation, suggesting that representations of individual fingers are highly overlapping within sensorimotor cortex. This is consistent with the view that finger representations are distributed across overlapping neuronal populations rather than localized to distinct peaks [5,6]. In the present study, ROI-based analyses centered on peak voxels were used to quantify condition-related changes in activation and to relate these to individuation performance. This approach was chosen to test hypothesis-driven effects at the level of common network recruitment, rather than to resolve fine-grained finger-specific representations. However, because finger-specific information is likely encoded in distributed voxel patterns, such ROI-based measures may have limited sensitivity to subtle differences between individual fingers. Future studies using approaches such as multivoxel pattern analysis or cluster-level metrics may be better suited to capture these distributed representations.

Our sample was limited to right-handed adults with normal hand function. However, the current study offers valuable background information and methodology that can be applied to broader clinical populations, including individuals with motor impairments or different hand dominance. The study population had a mean age over 60 years to allow for future comparisons with relevant patient groups.

## Conclusions

Simultaneous measurements of brain activity and finger kinematics revealed that lower finger independence was associated with increased recruitment of bilateral sensorimotor and ipsilateral cerebellar regions, both across fingers (i.e., differences relative to the thumb) and within fingers, reflecting inter- and intra-finger variability in the relationship between neural activity and finger individuation. These findings are consistent with greater engagement of distributed motor control networks during movements of less individuated fingers, possibly reflecting increased demands on coordination, sensorimotor integration, and movement control. The method is potentially viable for studies on motor control in persons suffering from impaired hand motor dexterity such as after a stroke, essential tremor or focal hand dystonia.

## Supporting information

S1 Table Group-level brain activation (n = 25 healthy right-hand–dominant older adults) for individual fingers vs rest (Finger > Rest) during Right finger movements. Results are FWE-corrected (p < 0.05). Regions within each cluster are categorized as contralateral/ipsilateral cortical regions or cerebellar regions (Neumorphometrics), excluding white matter voxels. “Voxels” indicates cluster size, “p” p-value for peak activation, “T Level” activation strength for peak activation, and MNI peak coordinates are given as “X”,“Y”, “Z”.
(PDF)

S2 Table Group-level brain activation (n = 25 healthy right-hand–dominant older adults) for individual fingers vs rest (Finger > Rest) during Left finger movements. Results are FWE-corrected (p < 0.05). Regions within each cluster are categorized as contralateral/ipsilateral cortical regions or cerebellar regions (Neumorphometrics), excluding white matter voxels. “Voxels” indicates cluster size, “p” p-value for peak activation, “T Level” activation strength for peak activation, and MNI peak coordinates are given as “X”,“Y”, “Z”.
(PDF)

## References

[1] Häger-Ross C, Schieber MH. Quantifying the independence of human finger movements: Comparisons of digits, hands, and movement frequencies. J Neurosci. 2000;20(22):8542–50. doi: 10.1523/JNEUROSCI.20-22-08542.2000 11069962 PMC6773164

[2] Johansson A-M, Grip H, Rönnqvist L, Selling J, Boraxbekk C-J, Strong A, et al. Influence of visual feedback, hand dominance and sex on individuated finger movements. Exp Brain Res. 2021;239(6):1911–28. doi: 10.1007/s00221-021-06100-0 33871660 PMC8277644

[3] Lang CE, Schieber MH. Human finger independence: Limitations due to passive mechanical coupling versus active neuromuscular control. J Neurophysiol. 2004;92(5):2802–10. doi: 10.1152/jn.00480.2004 15212429

[4] Xu J, Mawase F, Schieber MH. Evolution, biomechanics, and neurobiology converge to explain selective finger motor control. Physiol Rev. 2024;104(3):983–1020. doi: 10.1152/physrev.00030.2023 38385888 PMC11380997

[5] Ejaz N, Hamada M, Diedrichsen J. Hand use predicts the structure of representations in sensorimotor cortex. Nat Neurosci. 2015;18(7):1034–40. doi: 10.1038/nn.4038 26030847

[6] Schieber MH, Hibbard LS. How somatotopic is the motor cortex hand area?. Science. 1993;261:489–92. doi: 10.1126/science.8332915 8332915

[7] Berlot E, Prichard G, O’Reilly J, Ejaz N, Diedrichsen J. Ipsilateral finger representations in the sensorimotor cortex are driven by active movement processes, not passive sensory input. J Neurophysiol. 2019;121(2):418–26. doi: 10.1152/jn.00439.2018 30517048 PMC6397402

[8] Kolasinski J, Makin TR, Jbabdi S, Clare S, Stagg CJ, Johansen-Berg H. Investigating the stability of fine-grain digit somatotopy in individual human participants. J Neurosci. 2016;36(4):1113–27. doi: 10.1523/JNEUROSCI.1742-15.2016 26818501 PMC4728720

[9] Arbuckle SA, Weiler J, Kirk EA, Rice CL, Schieber M, Pruszynski JA, et al. Structure of population activity in primary motor cortex for single finger flexion and extension. J Neurosci. 2020;40(48):9210–23. doi: 10.1523/JNEUROSCI.0999-20.2020 33087474 PMC7687056

[10] Huber L, Finn ES, Handwerker DA, Bönstrup M, Glen DR, Kashyap S, et al. Sub-millimeter fMRI reveals multiple topographical digit representations that form action maps in human motor cortex. Neuroimage. 2020;208:116463. doi: 10.1016/j.neuroimage.2019.116463 31862526 PMC11829252

[11] Diedrichsen J, Wiestler T, Krakauer JW. Two distinct ipsilateral cortical representations for individuated finger movements. Cereb Cortex. 2013;23(6):1362–77. doi: 10.1093/cercor/bhs120 22610393 PMC3643717

[12] Belkacemi Z, van Dokkum LEH, Tchechmedjiev A, Lepetit-Coiffe M, Mottet D, Le Bars E. Can motion capture improve task-based fMRI studies of motor function post-stroke? A systematic review. J Neuroeng Rehabil. 2025;22(1):70. doi: 10.1186/s12984-025-01611-1 40181338 PMC11966795

[13] Van Dokkum LEH, Mottet D, Laffont I, Bonafé A, de Champfleur NM, Froger J, et al. Kinematics in the brain: Unmasking motor control strategies?. Exp Brain Res. 2017;235(9):2639–51. doi: 10.1007/s00221-017-4982-8 28573311 PMC5550544

[14] Casellato C, Ferrante S, Gandolla M, Volonterio N, Ferrigno G, Baselli G, et al. Simultaneous measurements of kinematics and fMRI: Compatibility assessment and case report on recovery evaluation of one stroke patient. J Neuroeng Rehabil. 2010;7:49. doi: 10.1186/1743-0003-7-49 20863391 PMC2955635

[15] Schieber MH. Individuated finger movements of rhesus monkeys: a means of quantifying the independence of the digits. J Neurophysiol. 1991;65(6):1381–91. doi: 10.1152/jn.1991.65.6.1381 1875247

[16] Lotze M. Performance control during longitudinal activation fMRI studies. Front Hum Neurosci. 2024;18:1459140. doi: 10.3389/fnhum.2024.1459140 39474131 PMC11521045

[17] Sanes JN, Donoghue JP. Plasticity and primary motor cortex. Annu Rev Neurosci. 2000;23:393–415. doi: 10.1146/annurev.neuro.23.1.393 10845069

[18] Maus B, Van Breukelen GJP, Goebel R, Berger MPF. Optimization of blocked designs in fMRI studies. Psychometrika. 2010;75:373–90. doi: 10.1007/s11336-010-9159-3

[19] Tzourio-Mazoyer N, Landeau B, Papathanassiou D, Crivello F, Etard O, Delcroix N, et al. Automated anatomical labeling of activations in SPM using a macroscopic anatomical parcellation of the MNI MRI single-subject brain. Neuroimage. 2002;15(1):273–89. doi: 10.1006/nimg.2001.0978 11771995

[20] Rolls ET, Huang C-C, Lin C-P, Feng J, Joliot M. Automated anatomical labelling atlas 3. NeuroImage. 2020;206:116189.doi: 10.1016/j.neuroimage.2019.116189 31521825

[21] Ashburner J. A fast diffeomorphic image registration algorithm. Neuroimage. 2007;38(1):95–113. doi: 10.1016/j.neuroimage.2007.07.007 17761438

[22] Oliveira ÍAF, van der Zwaag W, Raimondo L, Dumoulin SO, Siero JCW. Comparing hand movement rate dependence of cerebral blood volume and BOLD responses at 7T. Neuroimage. 2021;226:117623. doi: 10.1016/j.neuroimage.2020.117623 33301935

[23] Matsuo K, Chen S-HA, Tseng W-YI. AveLI: A robust lateralization index in functional magnetic resonance imaging using unbiased threshold-free computation. J Neurosci Methods. 2012;205(1):119–29. doi: 10.1016/j.jneumeth.2011.12.020 22233778

[24] Brett M, Anton JL, Valabregue R, Poline J-B. Region of interest analysis using the MarsBar toolbox for SPM 99. NeuroImage. 2002;:S497.

[25] Dayan E, Cohen LG. Neuroplasticity subserving motor skill learning. Neuron. 2011;72(3):443–54. doi: 10.1016/j.neuron.2011.10.008 22078504 PMC3217208

[26] Stefanescu MR, Thürling M, Maderwald S, Wiestler T, Ladd ME, Diedrichsen J, et al. A 7T fMRI study of cerebellar activation in sequential finger movement tasks. Exp Brain Res. 2013;228(2):243–54. doi: 10.1007/s00221-013-3558-5 23732948

[27] Haslinger B, Erhard P, Altenmüller E, Hennenlotter A, Schwaiger M, Gräfin von Einsiedel H, et al. Reduced recruitment of motor association areas during bimanual coordination in concert pianists. Hum Brain Mapp. 2004;22(3):206–15. doi: 10.1002/hbm.20028 15195287 PMC6871883

[28] Prati JM, Pontes-Silva A, Gianlorenço ACL. The cerebellum and its connections to other brain structures involved in motor and non-motor functions: A comprehensive review. Behav Brain Res. 2024;465:114933. doi: 10.1016/j.bbr.2024.114933 38458437

[29] Wang J, Sainburg RL. The dominant and nondominant arms are specialized for stabilizing different features of task performance. Exp Brain Res. 2007;178(4):565–70. doi: 10.1007/s00221-007-0936-x 17380323 PMC10702172

[30] Dexheimer B, Sainburg R. When the non-dominant arm dominates: The effects of visual information and task experience on speed-accuracy advantages. Exp Brain Res. 2021;239(2):655–65. doi: 10.1007/s00221-020-06011-6 33388816 PMC8063124

