# Supplementary material for: Brain activity related to individuated finger movement demonstrated by simultaneous motion capture and fMRI
Source: PLoS One. 2026 Aug 12;21(8):e0353360. doi: 10.1371/journal.pone.0353360 (PMC13465829; doi:10.1371/journal.pone.0353360)
Supplement: Supplementary file 1 [file pone.0353360.s001.pdf]

Table S1. Group-level brain activation (n = 25 healthy right-hand–dominant older adults) for individual fingers vs rest (Finger > Rest) during Right finger movements. Results are FWE-corrected ($p < 0.05$). Regions within each cluster are categorized as contralateral/ipsilateral cortical regions or cerebellar regions (Neumorphometrics), excluding white matter voxels. "Voxels" indicates cluster size, "p" p-value for peak activation, "T Level" activation strength for peak activation, and MNI peak coordinates are given as "X","Y", "Z".

Finger (Right)	Contralateral (Left) regions	Ipsilateral (Right) regions	Cerebellar regions	Voxel	T Level	p	X	Y	Z
Thumb > Rest	Postcentral gyrus (25.2%), precentral gyrus (14.2%), supramarginal gyrus (9.9%), superior parietal lobule (8.2%), supplementary motor cortex (7.3%), parietal operculum (4.6%), central operculum (1.7%), planum temporale (1.3%), precentral gyrus medial segment (1.2%), superior frontal gyrus (0.8%)	Supplementary motor cortex (4.5%), superior frontal gyrus (0.2%)		8043	14,3	0,00	-36	-20	57
		Occipital fusiform gyrus (2.2%), fusiform gyrus (0.7%)	Right Cerebellum Exterior (84.6%)	2691	10,6	0,00	23	-56	-24
		Precentral gyrus (84.8%), postcentral gyrus (0.2%)		468	9,9	0,00	59	3	39
		Precentral gyrus (96.2%),Postcentral gyrus (1.5%)		132	6,6	0,00	44	-11	56
	Precentral gyrus (83.1%), postcentral gyrus (0.3%)			372	6,3	0,00	-59	3	30
		Supramarginal gyrus (54.5%), Postcentral gyrus (33.3%)		132	5,3	0,00	54	-20	36
		Planum temporale (55.3%), Superior temporal gyrus (31.9%), Supramarginal gyrus (4.3%)		47	5,2	0,00	63	-32	18
		Inferior temporal gyrus (81.7%), inferior occipital gyrus (8.5%), mid temporal gyrus (1.4%)		71	5,1	0,00	57	-66	-9
			Left Cerebellum Exterior (100.0%)	18	4,8	0,00	-24	-62	-24
		Central operculum (54.5%)		11	4,7	0,00	57	-17	20
Index vs Rest	Postcentral gyrus (26.3%), precentral gyrus (17.0%), supplementary motor cortex (9.5%), superior parietal lobule(8.2%), supramarginal gyrus (8.0%), precentral medial (2.6%), superior frontal (1.8%), middle cingulate (0.3%)	Supplementary motor cortex (6.4%), superior frontal gyrus (0.4%)		7220	15,0	0,00	-38	-21	59
			Right Cerebellum Exterior (85.3%)	2011	11,2	0,00	21	-56	-23
		Precentral gyrus (87.7% Right), postcentral gyrus (0.3%)		397	8,8	0,00	59	3	39
	Precentral gyrus (87.6%)			314	6,3	0,00	-56	-2	44
		Precentral gyrus (91.0%), postcentral gyrus (0.7%)		134	6,2	0,00	45	-9	57
	Parietal operculum (69.1% Left)			123	5,7	0,00	-48	-27	20
	Planum temporale (39.7%), Supramarginal gyrus (19.1%), Parietal operculum (11.8%)			68	5,3	0,00	-48	-44	23
		Supramarginal gyrus (62.5%), postcentral gyrus (35.4%)		48	4,9	0,00	56	-20	39
		Superior temporal gyrus (94.7%), planum temporale (5.3%)		19	4,9	0,00	65	-32	17
		Precentral gyrus (37.5%)		16	4,9	0,00	56	6	21
			Left Cerebellum Exterior (100.0%)	24	4,8	0,00	-26	-62	-26
	Putamen (100%)			15	4,8	0,00	-26	2	9
Middle vs Rest	Postcentral gyrus (17.5%), precentral gyrus (16.3%), supramarginal gyrus (10.2%), superior parietal lobule(9.1%), supplementary motor cortex (5.9%), parietal operculum (3.9%), planum temporale (2.5%), central operculum (2.5%), precentral gyrus (1.7%), superior frontal gyrus (1.7%), middle cingulate gyrus (0.4%)	Supplementary motor cortex (5.0%), superior frontal gyrus (1.1%), middle cingulate gyrus (0.2%)		12915	16,3	0,00	-38	-21	59
		Fusiform gyrus (0.5%),occipital fusiform gyrus (0.2%)	Right Cerebellum Exterior (77.4%)	3282	13,4	0,00	21	-54	-24
		Precentral gyrus (61.0%), inferior frontal gyrus (9.1%), central operculum (1.4%), frontal operculum (1.3%), postcentral gyrus (0.4%)		1839	10,5	0,00	59	5	38
		Supramarginal gyrus (39.0%), postcentral gyrus (15.1%), superior parietal lobule (7.6%), planum temporale (5.1%), parietal operculum (4.6%), central operculum (4.3%), superior temporal gyrus (3.8%)		2761	6,9	0,00	65	-32	18
	Occipital fusiform gyrus (0.7%)		Left Cerebellum Exterior (96.6%)	860	6,5	0,00	-24	-62	-26
			Right Cerebellum Exterior (100.0%)	1	4,8	0,00	12	-69	-45
		Inferior temporal gyrus (100%)		12	4,8	0,00	59	-63	-9
			Right Cerebellum Exterior (75.0%)	4	4,8	0,00	8	-72	-44
Ring vs Rest	Postcentral gyrus (19.3%), precentral gyrus (16.5%), supramarginal gyrus (9.3%), superior parietal lobule(7.6%), supplementary motor cortex (7.3%), parietal operculum (4.6%), planum temporale (3.0%), precentral gyrus (2.8%), central operculum (1.4%), superior frontal gyrus (1.2%), middle cingulate gyrus (0.5%)	Supplementary motor cortex (4.9%), superior frontal gyrus (0.4%)		10447	15,3	0,00	-38	-23	59
			Right Cerebellum Exterior (80.7%)	2336	12,1	0,00	21	-54	-24
		Precentral gyrus (79.4%)		709	10,0	0,00	59	3	39
		Precentral gyrus (67.7%), postcentral gyrus (0.5%)		189	6,3	0,00	44	-9	57
		Supramarginal gyrus (28.8%), postcentral gyrus (12.6%), central operculum (11.5%), planum temporale (10.5%), sup. temporal gyrus (9.6%), parietal operculum (4.8%)		1144	6,3	0,00	65	-32	18
			Left Cerebellum Exterior (99.0%)	399	6,1	0,00	-26	-60	-26
	Central operculum (78.6%), precentral gyrus (2.4%)			84	5,0	0,00	-44	-3	12
		Central operculum (50.0%)		2	4,7	0,00	45	2	14
	Putamen (100%)			2	4,6	0,00	-26	2	11
Little vs Rest	Precentral gyrus (11.8%), postcentral gyrus (11.0%), supramarginal gyrus (9.5%), superior parietal lobule (9.1%), supplementary motor cortex (4.0%), parietal operculum (3.7%), central operculum (3.7%), planum temporale (2.6%), precentral gyrus (1.7%), superior frontal gyrus (1.6%), anterior insula (0.8%), middle cingulate gyrus (0.5%), sup. temporal gyrus (0.3%), planum polare (0.2%)	Precentral gyrus (7.8%), supplementary motor cortex (3.7%), superior frontal gyrus (1.7%), inferior frontal gyrus (1.4%), central operculum (1.3%), anterior insula (0.5%), precentral gyrus (0.3%), frontal operculum (0.2%), middle cingulate gyrus (0.2%)		20817	16,6	0,00	-36	-21	59
		Fusiform gyrus (0.5%)	Right Cerebellum Exterior (75.8%), Left Cerebellum Exterior (0.2%)	4563	14,5	0,00	23	-54	-24
		Supramarginal gyrus (40.9%), postcentral gyrus (14.9%), superior parietal lobule(6.9%), parietal operculum (6.5%), planum temporale (5.0%), central operculum (4.2%), superior temporal gyrus (3.4%)		4635	8,0	0,00	59	-18	20
			Left Cerebellum Exterior (98.5%)	1648	7,9	0,00	-24	-62	-24
				390	6,2	0,00	-56	-72	-3
	Inferior occipital gyrus (45.9%), middle temporal gyrus (39.0%), inferior temporal gyrus (10.5%)	Inferior temporal gyrus (68.4%), middle temporal gyrus (23.8%)		206	5,6	0,00	59	-63	-6
	Superior parietal lobule (100%)			2	4,7	0,00	-33	-60	57
	Middle frontal gyrus (60%)			20	4,7	0,00	-39	39	24
