# Supplementary material for: Brain activity related to individuated finger movement demonstrated by simultaneous motion capture and fMRI
Source: PLoS One. 2026 Aug 12;21(8):e0353360. doi: 10.1371/journal.pone.0353360 (PMC13465829; doi:10.1371/journal.pone.0353360)
Supplement: Supplementary file 2 [file pone.0353360.s002.pdf]

Table S2. Group-level brain activation (n = 25 healthy right-hand–dominant older adults) for individual fingers vs rest (Finger > Rest) during Left finger movements. Results are FWE-corrected ($p < 0.05$). Regions within each cluster are categorized as contralateral/ipsilateral cortical regions or cerebellar regions (Neumorphometrics), excluding white matter voxels. "Voxels" indicates cluster size, "p" p-value for peak activation, "T Level" activation strength for peak activation, and MNI peak coordinates are given as "X","Y", "Z".

Finger (Left)	Contralateral (Right) regions	Ipsilateral (Left) regions	Cerebellar regions	Voxel	TLevel	p	X	Y	Z
**Thumb vs Rest**	Postcentral gyrus (23.0%), Precentral gyrus (18.8%), Superior parietal lobule (7.3%), SMC supplementary motor cortex (7.1%), Supramarginal gyrus (6.8%), Parietal operculum (1.7%), Planum temporale (1.6%), Central operculum (1.4%), Precentral gyrus (1.2%), Superior frontal gyrus (0.7%)	SMC supplementary motor cortex (6.1%), Precentral gyrus medial segment (0.6%)		8310	14,0	0,00	41	-15	56
			Left Cerebellum Exterior (85.2%)	1770	10,1	0,00	-21	-57	-24
		Precentral gyrus (64.1%), Postcentral gyrus (2.7%)		412	7,9	0,00	-39	-15	57
		Precentral gyrus (80.9%)		335	6,9	0,00	-59	3	26
		Supramarginal gyrus (64.9%), Postcentral gyrus (35.1%)		134	5,4	0,00	-57	-24	42
	Putamen (87.5%), Pallidum (12.5%)			8	4,8	0,00	24	0	-6
**Index vs Rest**	Postcentral gyrus (29.2%), Precentral gyrus (20.4%), SMC supplementary motor cortex (10.8%), Superior parietal lobule (3.0%), Supramarginal gyrus (3.0%), Superior frontal gyrus (0.9%), Precentral gyrus (0.3%), Middle cingulate gyrus (0.2%)	SMC supplementary motor cortex (9.3%), Precentral gyrus medial segment (0.4%)		5293	14,2	0,00	41	-18	54
			Left Cerebellum Exterior (83.5%)	1104	9,7	0,00	-18	-57	-24
	Precentral gyrus (91.8%), Postcentral gyrus (0.4%)			257	9,0	0,00	59	2	41
		Precentral gyrus (67.6%), 1.3% Postcentral gyrus (67.6%		309	7,2	0,00	-39	-15	57
		Precentral gyrus (79.2%)		168	6,4	0,00	-59	2	26
	Central operculum (63.6%)			33	5,2	0,00	57	-17	20
	Supramarginal gyrus (100%)			2	4,9	0,00	57	-26	51
	Supramarginal gyrus (100%)			1	4,7	0,00	56	-27	53
**Middle vs Rest**	Postcentral gyrus (20.2%), Precentral gyrus (15.6%), SMC supplementary motor cortex (9.1%), Supramarginal gyrus (9.1%), Superior parietal lobule (7.6%), Superior frontal gyrus (1.4%), Precentral gyrus (1.1%), Middle cingulate gyrus (0.7%)	SMC supplementary motor cortex (8.3%), Precentral gyrus medial segment (1.2%), Superior frontal gyrus (1.0%), Middle cingulate gyrus (0.2%)		8363	15,3	0,00	38	-23	51
			Left Cerebellum Exterior (72.9%)	2594	11,8	0,00	-18	-56	-24
	Precentral gyrus (78.0%), OpIFG opercular part of the inferior frontal gyrus (2.9%)			1074	9,9	0,00	59	3	39
		Precentral gyrus (56.3%), Postcentral gyrus (1.6%)		568	8,8	0,00	-39	-14	59
		Precentral gyrus (46.6%), Central operculum (16.1%)		930	8,1	0,00	-59	3	24
			Right Cerebellum Exterior (98.5%)	401	6,4	0,00	23	-60	-24
	Planum temporale (21.8%), Parietal operculum (19.5%), Central operculum (18.0%), Superior temporal gyrus (8.0%), Supramarginal gyrus (7.4%)			473	6,4	0,00	56	-20	20
	Putamen (45.1%), anterior insula (10.2%)			215	5,5	0,00	35	0	-3
		Postcentral gyrus (35.1%), Supramarginal gyrus (8.8%)		57	5,1	0,00	-56	-23	24
		Supramarginal gyrus (78.3%), Postcentral gyrus (21.7%)		92	5,1	0,00	-53	-27	41
	Pallidum (40%)			5	5,0	0,00	23	-5	-8
		Putamen (42.9%)		14	4,7	0,00	-32	-2	6
**Ring vs Rest**	Postcentral gyrus (21.8%), Precentral gyrus (17.6%), SMC supplementary motor cortex (9.8%), Superior parietal lobule (5.8%), Supramarginal gyrus (5.4%), Precentral gyrus medial segment (1.6%), Superior frontal gyrus (1.1%), Middle cingulate gyrus (0.9%)	SMC supplementary motor cortex (8.7%), Precentral gyrus medial segment (1.2%), Superior frontal gyrus (1.1%), Middle cingulate gyrus (0.1%), Precentral gyrus (0.1%)		6869	14,6	0,00	39	-26	62
			Left Cerebellum Exterior (84.1%)	1450	10,2	0,00	-20	-56	-24
	87.2% Precentral gyrus (87.2%), Postcentral gyrus (0.7%)			413	10,2	0,00	59	3	39
		Precentral gyrus (56.8%), Central operculum (10.2%), Postcentral gyrus (1.0%), anterior insula (0.3%)		1396	8,1	0,00	-38	-15	59
	Central operculum (30.3%), Parietal operculum (21.6%), Planum temporale (20.6%), Supramarginal gyrus (5.2%), Superior temporal gyrus (1.4%), Postcentral gyrus (0.2%)			422	6,8	0,00	57	-18	18
	Putamen (80.7%), Pallidum (9.1%)			88	5,5	0,00	29	-6	-3
		Supramarginal gyrus (74.7%), Postcentral gyrus (25.3%)		83	5,4	0,00	-57	-26	44
	Precentral gyrus (56.7%)			30	5,1	0,00	57	6	20
		Putamen (37.5%)		16	4,9	0,00	-32	-3	6
**Little vs Rest**	Precentral gyrus (14.4%), Postcentral gyrus (12.2%), Supramarginal gyrus (8.7%), Superior parietal lobule (6.3%), SMC supplementary motor cortex (4.9%), Precentral gyrus medial segment (2.3%), Parietal operculum (2.3%), Superior frontal gyrus (2.0%), Planum temporale (1.6%), Central operculum (1.4%), Middle cingulate gyrus (0.9%), Superior temporal gyrus (0.4%)	Precentral gyrus (9.7%), SMC supplementary motor cortex (4.5%), Precentral gyrus (1.2%), Superior frontal gyrus (1.1%), Postcentral gyrus (0.6%), Central operculum (0.6%), Middle cingulate gyrus (0.3%)		16698	16,3	0,00	39	-26	62
			Left Cerebellum Exterior (82.0%)	2491	11,6	0,00	-21	-54	-26
		Supramarginal gyrus (43.0%), Postcentral gyrus (26.9%), Parietal operculum (5.2%), Central operculum (1.2%)		1699	7,3	0,00	-54	-27	39
			Right Cerebellum Exterior (98.7%)	479	6,4	0,00	24	-62	-26
	Central operculum (61.7%)			47	5,3	0,00	45	2	14
		Superior parietal lobule (100%)		60	5,2	0,00	-38	-48	63
		Planum temporale (50.0%), Supramarginal gyrus (11.8%), Parietal operculum (8.8%)		34	5,0	0,00	-48	-44	24
		Superior parietal lobule (83.7%)		43	4,9	0,00	-38	-39	54
		Superior parietal lobule (100%)		6	4,8	0,00	-20	-57	71
	Superior parietal lobule (100%)			4	4,8	0,00	17	-53	72
	Putamen (100%)			4	4,7	0,00	29	-6	-3
	Anterior insula (100%)			1	4,6	0,00	35	2	9
